# Mechanobiology in Action: Biomaterials, Devices, and the Cellular Machinery of Force Sensing

**DOI:** 10.3390/biom15060848

**Published:** 2025-06-10

**Authors:** Miriam Lucariello, Maria Luisa Valicenti, Samuele Giannoni, Leonardo Donati, Ilaria Armentano, Francesco Morena, Sabata Martino

**Affiliations:** 1 Department of Chemistry, Biology and Biotechnology, Biochemical and Biotechnological Sciences, University of Perugia, 06122 Perugia, Italy; miriam.lucariello@unipg.it (M.L.); marialuisa.valicenti@dottorandi.unipg.it (M.L.V.); samuele.giannoni@dottorandi.unipg.it (S.G.); leonardo.donati@unipg.it (L.D.); 2 Department of Economics, Engineering, Society and Business Organization (DEIM), UdR INSTM, University of Tuscia, Largo dell’Università snc, 01100 Viterbo, Italy; ilaria.armentano@unitus.it

**Keywords:** mechanotransduction, mechanosensing, stem cells, differentiation, biomaterials, 3D printing, biodevices, roughness, stiffness, YAP/TAZ, cell metabolism, organelles

## Abstract

Mechanical forces are increasingly recognised as fundamental regulators of cellular function, complementing classical biochemical cues to direct development, tissue homeostasis, and disease progression. Cells detect external and internal forces via mechanosensor proteins and adapt their cytoskeletal architecture, leading to changes in cell behaviour. Biomaterials and biodevices come to the aid of tailoring biomaterials’ properties in terms of chemical/physical properties and, by emulating dynamical forces, e.g., shear stress and cell swelling, they may enlighten mechanobiological processes. Additionally, emerging technologies expand the experimental toolkit for probing mechanobiological phenomena in complex, customisable settings. Central to these processes are mechanotransducer proteins and membrane–organelle networks that convert mechanical deformation into biochemical signals, orchestrating downstream transcriptional and post-translational modifications. This review highlights how through bridging material engineering and cellular mechanics, mechanobiology provides a unified framework to understand how physical forces shape tissues and drive pathologies. The continued integration of advanced biomaterials, dynamic biodevices, and multiscale analytical methods promises to uncover new mechanistic insights and inform the development of mechanotherapeutic strategies.

## 1. Feeling the Force: How to Mimic External Stimuli

Biomedical research has primarily focused on disease, development and tissue homeostasis by studying the biochemical nature, overlooking other equally important aspects. Among these, the response to mechanical stimuli plays a crucial role in various processes, including tissue patterning during the development of balancing forces as well as in maintaining homeostasis and preventing cancer metastasis [1,2,3]. Over time, the interest in the studies of cellular mechanics gave rise to a new and growing field that attracts researchers from cell biology, biochemistry, bio- and tissue engineering, and biophysics, which is known as ‘mechanobiology’ [4]. The field of mechanobiology is not only focused on the biomechanical properties of an organism conditioned by external stimuli but also involves understanding information about the processing and phenotypic change due to a mechanical stimulus [5]. Nowadays, it is well established that cells continuously sense and process information from their surrounding microenvironment to regulate their behaviour both at the tissue and organismal level [6,7]. Thanks to this ability, cells are capable of altering their internal architecture and composition, leading to various biological responses, including differentiation, proliferation, growth, metabolic processes, and migration [8]. For instance, mechanical cues—especially surrounding the characteristics of cells (e.g., substrate stiffness) and ECM topography—drive human mesenchymal stem cell (hMSC) differentiation towards osteogenic lineages and facilitate bone tissue regeneration.

**Figure 1:**
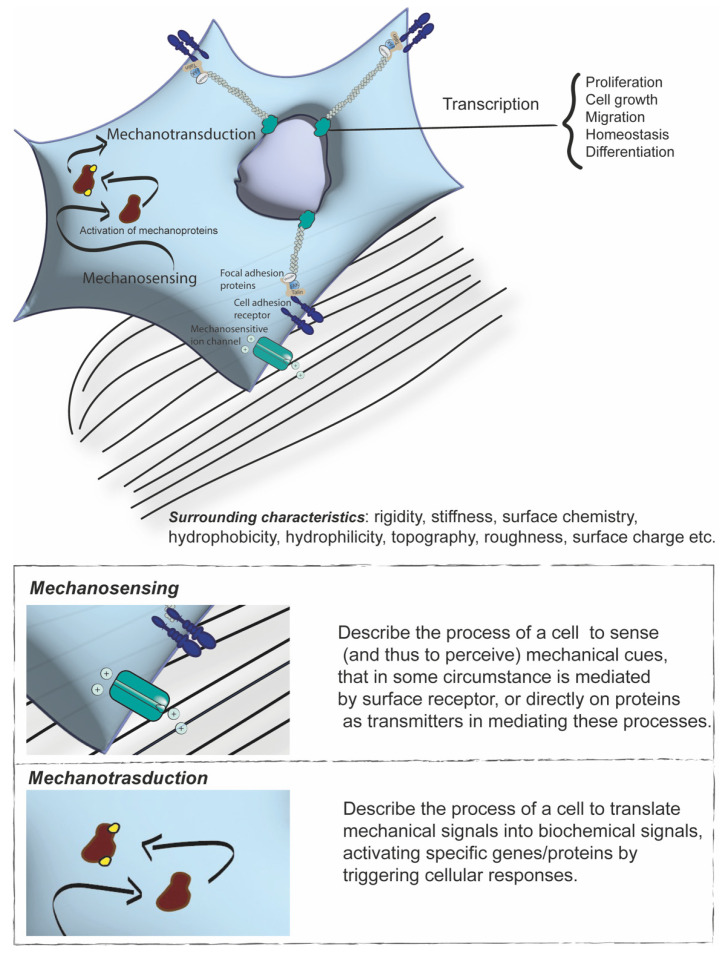
The schematic illustrates the definition and the differences between mechanosensing and mechanotransduction. The cartoon illustrates how mechanical cues are perceived through mechanosensor proteins that transmit information into the cell, resulting in the activation of mechanotransduction pathways and consequent downstream effects on transcriptional activity and cellular behaviour.

Thus, a more comprehensive framework for understanding cellular functions is needed. While the study of biochemical pathways remains essential, it is equally important to consider the mechanical effects exerted on cells [9,10]. These two aspects are closely interconnected. Indeed, the propagation of mechanical forces is regarded as one of the fastest signalling pathways, thus enabling the rapid conversion of physical stimuli into biochemical signals and explaining the ability of cells to adapt to their surrounding milieu rapidly [11,12,13]. Cells experience mechanical forces either externally, such as shear stress caused by blood flow, the composition of the extracellular matrix (ECM), or the properties of neighbouring cells, or internally, as they generate forces to reshape ECM or remodel tissues during embryonic development [2,6,14,15,16]. Remarkably, cells have developed the ability to sense forces provided by their external environment—called mechanosensing—through proteins and receptors that detect these mechanical inputs and convert them into signalling events, triggering specific biological responses. This process is known as mechanotransduction (Figure 1) [8,17,18,19,20]. In more detail, cells integrate mechanical and chemical signals into biological responses, thereby regulating gene expression through transcriptional and epigenetic mechanisms [18,21,22]. In response to these cues, cells generate contractile forces via cytoskeletal networks (i.e., microtubules, F-actin, and intermediate filaments) associated with actin-linking proteins (i.e., myosin II, cofilin, and filamin), creating internal tension [23,24]. Importantly, cells sense surrounding characteristics (Figure 1), such as stiffness, roughness, surface chemistry, and topographical cues (e.g., curvature, roughness/smoothness, micro/nanopatterns, and hydrophilicity/hydrophobicity) that allow directing downstream signalling and gene expression programs that control cell differentiation, migration, and function [25,26,27].

Cells can detect mechanical signals through various mechanisms: mechanical forces can directly or indirectly modulate ion channels (e.g., through the mechanosensitive ion channel Piezo1) [28], or transmembrane proteins [7,29,30]; ECM may vary in its stiffness and viscoelasticity but also in geometrical organisation, roughness, curvatures, and fibrosity [8,13,14,31,32,33,34]. Additionally, key components of mechanosensing may include cell-surface receptors that mediate interplay with the ECM or neighbouring cells. In this context, contact inhibition, despite its historical name, is a consequence of mechanical cell confinement to a small area due to cell crowding, which reduces both proliferation and cell growth [35,36,37]. This process is crucial for tissue development and homeostasis as well as for preventing uncontrolled cell proliferation. In this context, integrins at focal adhesions and cadherins at adherens junctions play critical roles by linking to the actin cytoskeleton, thereby facilitating the transmission of mechanical signals into the cell [38]. For instance, studies have shown that hMSCs exhibit “mechanical memory”, retaining information from previous mechanical stimuli to guide future behaviour [39,40,41,42]. The effects of intermittent stretching on various cellular properties were observed through transcriptional and metabolic analyses, resulting in a mechanical memory that enables cells to carry out specific biological responses [43]. Particularly, intermittent stretching was observed in cytoskeletal and nuclear remodelling, finding that cells preserve mechanical memory by highlighting the role of protein histone H3 modification in mechanical memory-mediated smooth muscle differentiation [11,40]. Moreover, it was also demonstrated how substrate stiffness influences the activation of mechanoproteins, which regulate cell fate, and how “mechanical dosing” can induce mechanical memory that drives the osteogenic differentiation of hMSCs [44].

Given that external stimuli influence cellular properties, it becomes necessary to adopt new strategies to study mechanotransduction, as in the conventional two-dimensional (2D) cell culture, the cells adhere to a flat surface, typically a petri dish of glass or polystyrene, that does not replicate the complexity of cellular microenvironments in both physiologic and pathophysiologic states [45]. Moreover, physical signals can undergo dynamic changes during processes such as development, tissue repair, or pathological contexts [46], and they do not provide meaningful information regarding the fundamental dynamics that living cells and tissues experience [47]. Replicating the dynamic, multicellular mechanical environment of native tissues remains a significant challenge in vitro, as current platforms often fail to fully capture the complex spatial and temporal heterogeneity of mechanical cues present in vivo [48,49]. To address these gaps, researchers should develop materials and/or systems that better mimic the heterogeneous and dynamic nature of living tissues [48].

**Figure 2:**
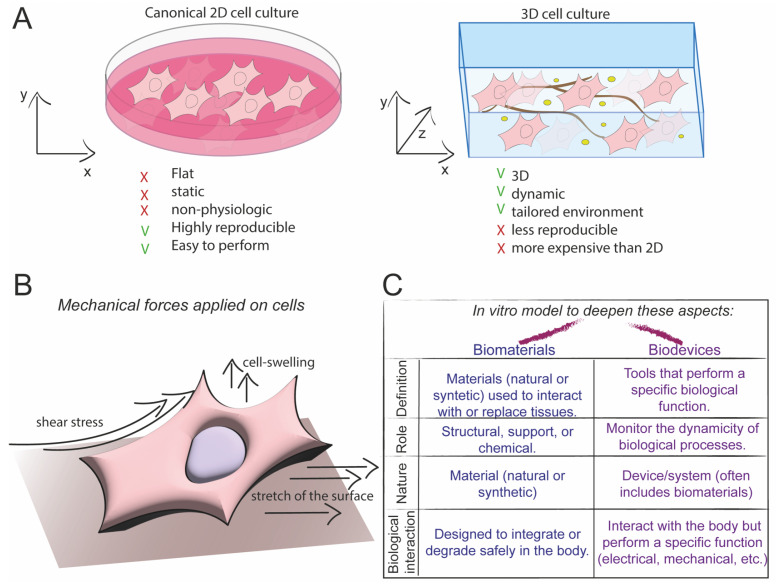
Schematic representation of different mechanical properties applied to cells. (**A**) Differences between classical 2D cell culture and 3D cell culture. (**B**) Representation of mechanical forces applied to cells and subsequent models to explore them. (**C**) The scheme represents differences between biomaterials and biodevices.

Recent research has shifted towards 3D cell culture, which involves cultivating cells within a supporting scaffold (e.g., hydrogel-like Matrigel) (Figure 2A). In this context, organoids and spheroids represent advancements in 3D cell culture, as they closely recapitulate the structural and functional complexity of native tissues. Their use enhances the physiological relevance of in vitro models, making them indispensable tools for disease modelling, drug screening, and regenerative medicine research. However, 3D models also present several limitations. The inability to faithfully mimic the complexity of native extracellular matrix (ECM) microarchitecture, including fibre network topology and viscoelastic properties, limits the accuracy of mechanobiological investigations.

Cells in vivo experience confined mechanical constraints and dynamic remodelling of their microenvironment, which are difficult to reproduce in static or simplified 3D culture systems [48,49]. Furthermore, scalability is still a major drawback: although 3D structures and organoids provide more dimensionality, their complex manufacturing procedures frequently make it impossible to perform the high-throughput screening required for large-scale research and drug discovery [50]. These systems often lack the spatial resolution to recapitulate the hierarchical matrix architecture and dynamic mechanical gradients inherent in native tissues, leading to diminished physiological relevance [51]. It became necessary to balance the model complexity and practicality to achieve scalable, physiologically relevant platforms that truly mirror the in vivo mechanical milieu. Moreover, the different mechanical stimuli sensed by cells (Figure 2B) and the interaction between cells and the ECM are further elucidated through various approaches that have contributed to the advancement of knowledge in mechanobiology, including biomaterials and biodevices (Figure 2C), which will be discussed in detail in Section 2.

## 2. Biomaterials and Biodevices May Mimic ECM Topographical and Mechanical Properties

The impact of mechanical cues in regulating cellular behaviour and dysregulated mechanotransduction plays a pivotal role in the progression of various diseases, including cancer, fibrosis, cardiovascular disorders, and neurodegenerative diseases [12,52].

A fundamental question remains: how do cells integrate diverse biomechanical and mechanical cues into coordinated biological responses? Here comes the aid of biomaterials and biodevices, which proved to be essential tools to address this challenge: through the modulation of biomaterial characteristics, it is possible to analyse the downstream effects on cells; similarly, by modulating the external forces applied to cells, it becomes possible to mimic the dynamic physiological and pathophysiological processes (Figure 2C).

### 2.1. Biomaterials

The field of biomaterials has made dramatic advances in the last decades, leading to the development of complex material systems. Such progress is due to the capability to engineer different physicochemical properties, providing distinct manipulative cues for mammalian cells. In general, biomaterials involved in biomedical applications may be both natural and synthetic polymers, each one with an advantage: natural polymers are recognised as self by cells and tissues, while synthetic polymers are customisable in composition and thus feature multi-skilled mechanical properties [53,54].

Considering that our body’s cells and tissues are constantly exposed to various physical signals, it is desirable to develop in vitro techniques that isolate different aspects of mechanical cues and examine how these aspects regulate the phenotypic and functional cellular responses to mechanical stimuli [55]. Due to the complexity of these processes, a fundamental element of biomaterial design is determining which factor should be prioritised to analyse a specific biological response [17] (Table 1).

#### 2.1.1. Hydrogels

Hydrogels are 3D structures of crosslinked hydrophilic polymer networks, allowing the encapsulation of live cells in a water-swollen environment [94,95,96,97]. Through chemical modifications, it is possible to tune mechanical properties and control their biological effect [94,95,98,99,100]. For example, increasing the crosslink densities in hydrogel increases stiffness but also changes the behaviour of adhesive ligands, substrate porosity, and viscoelastic properties [101,102]. Hydrogel can serve as a highly regulated replica of the native ECM [103]. For instance, hydrogels with defined rigidities have demonstrated that hMSCs act as mechanical “chameleons”, adopting fates corresponding to the stiffness of their native tissues. Indeed, hMSCs differentiate into adipocytes on soft, fat-like substrates; neurones on brain-like stiffness; muscle on intermediate stiffness; and bone on the stiffest substrates. Prouvè et al. demonstrated that human hMSCs differentiation can be directed towards osteoblasts and even osteocytes on hydrogels [104].

#### 2.1.2. Film

Films are thin, flexible materials that can be engineered to mimic the mechanical properties of native tissues, thereby influencing cellular behaviours such as adhesion, proliferation, and differentiation. For instance, composite films made from poly(vinylidene fluoride-trifluoroethylene) and barium titanate nanoparticles have demonstrated enhanced piezoelectric properties, which, when combined with ultrasound stimulation, promote neuronal differentiation in SH-SY5Y neuroblastoma cells [105,106,107]. Furthermore, co-polymer films PCL-PEG-PCL with adjustable mechanical and degradation properties have been created as a result of developments in polymer science, which makes them appropriate for tissue engineering applications [108,109]. These advancements highlight the potential of film biomaterials as dynamic interfaces that actively modify cellular responses through mechanistic cues while also promoting tissue regeneration.

#### 2.1.3. Scaffolds

Scaffolds are a class of 3D biomaterials with a defined structure, and the porosity can be modulated to allow for cell migration into the scaffold, allowing its application in regenerative and/or therapeutic medicine fields [110]. The rigidity of biomaterials may contribute to different pathophysiological contexts thanks to their characteristics, such as porosity, rigidity, and degradability. In some contexts, scaffolds may facilitate alignment and enable cell infiltration and nutrient transfer [111,112]. In regenerative medicine, scaffolds are employed in creating channel-like structures, e.g., for collagen fibres, thus providing an environment conductive to the formation of aligned myofibres [113,114,115]. hMSCs preferentially differentiate into osteoblasts on stiffer substrates, while flexibility promotes adipogenic differentiation [116,117,118,119]. One notable 3D biomaterial that has progressed towards clinical application is a bioresorbable coronary artery scaffold (stent) engineered to provide temporary vascular support and deliver therapeutic agents. Early data demonstrated sustained safety over time [120]; however, more recent findings reported increased target lesion failure and thrombosis in Absorb-BVS-treated patients [121].

#### 2.1.4. Smart Biomaterials

Recently, an emerging group of biomaterials, in addition to replicating the ECM, is also capable of changing structure according to their environment and external stimuli; these are known as smart biomaterials. These differ from canonical biomaterials in the ability to dynamically alter their physical and chemical properties in both space and time. Specifically, active biomaterials can be engineered to respond to external stimuli (e.g., light, heat, magnetic fields, pH, enzymes), thereby enabling the dynamic modulation of the mechanical properties of the cellular microenvironment [101,122,123,124]. Smart biomaterials offer unprecedented opportunities to investigate cellular behaviours under physiological conditions. For instance, photosensitive hydrogels incorporate photolabile molecules that undergo chemical modifications upon exposure to light, thus leading to changes in material stiffness: it has been demonstrated that crosslinked polymers functionalised with o-nitrobenzyl alcohol derivatives can be softened through light exposure, while crosslinking methacrylate chains through photoinduction increases rigidity [125]. These innovative tools can recapitulate the dynamic substrate stiffness, such as in fibrosis [122]. Another interesting class of smart biomaterials consists of thermoresponsive hydrogels, such as those based on poly(N-isopropyl acrylamide) and its copolymers, which can alter their mechanical properties in response to temperature fluctuations. They can contract or expand in response to temperature changes, resulting in an increase or decrease in stiffness, respectively. These systems are ideal for studying how temperature-dependent changes in the microenvironment influence cellular behaviour [126,127,128,129,130]. Mechanical properties may be customised through reversible chemical bonds, i.e., by cyclodextrin or adamantane, that can reduce or increase crosslinking density [118,131,132]. To this aim, Soriente et al. developed bioactive films that release β-glucans and a large number of proteins for wound healing and the regeneration of injured tissues [133] (Table 1).

#### 2.1.5. Physical/Chemical Properties of Biomaterials

Beyond the structure of biomaterials, other characteristics should be considered. For instance, the topography (surface features or structures present on the material at the micro- and nanoscale) and the roughness (degree of surface irregularity or texture of a biomaterial) should also be considered. Advances in research on biomaterials have enabled the investigation of physical parameters beyond stiffness, such as the topological features of substrates that can influence cell behaviour in different contexts [134]. Surface roughness influences the expression of osteogenic factors like alkaline phosphatase (ALP) and collagen type 1 (COL1) and mineralisation in hMSCs cultured in deprived osteogenic factors [25,26]. Additionally, topography plays a significant role in other biomedical applications; for instance, specific a-C:H nanopatterns have been shown to function as mechanotransducers that induce the neuronal differentiation of hBM-MSCs in vitro even in the absence of biochemical cues [135,136].

Moreover, surface chemical properties such as electrophilicity, hydrophobicity, and hydrophilicity are also crucial in influencing cell–material interactions [137,138]. Hydrophilic surfaces generally promote better cell adhesion and spreading compared to hydrophobic ones [139]. Electrophilic surfaces can further modulate cellular responses by interacting with nucleophilic biomolecules at the cell membrane, influencing cell signalling pathways and tissue integration [140]. Therefore, optimising the surface chemistry in tandem with physical characteristics, such as topography and roughness, is essential for the design of next-generation biomaterials.

### 2.2. Micro- and Nanocomposites

Biocomposites are a class of biomaterials matrix composed of phases of micro- or nano-sized dimensions [141] (Table 1). The polymer nanocomposite approach has emerged in the last decade as an efficient method to finely modulate the mechanical, electrical, thermal, and morphological properties of polymer matrices by introducing specific nanomaterials [142,143]. This approach could significantly advance the development of biomaterials with tuneable physical properties designed to elicit targeted biological responses. Whereas traditional biomaterials typically perform a single primary function, the ultimate properties of biocomposites depend on the chosen biomaterials’ physicochemical characteristics, morphology, aspect ratio, weight fraction, and dispersion method. For instance, hydroxyapatite is used to induce osteoconductive properties and modulate the morphology and mechanical properties of the membranes [144], carbon nanotubes improve the electrical and dielectric properties of the polymer matrices and affect the electrical stimulation [145,146,147,148], and silver nanoparticles can induce antibacterial properties in the polymer without inducing toxic effects in the cells [149,150,151,152]. Using cellulose nanocrystals as a sustainable additive, also at low content (5 wt.%), can affect the mechanical properties of the polymer, increasing Young’s modulus values and maintaining high values of elongation at break [153,154]. However, the improvement and, in general, modulation in mechanical properties depend on several factors, such as the type of polymer matrix, the kind of fillers, the weight content, and, overall, the presence or lack of intimate adhesion between the filler and the matrix. The mechanical properties of these materials, such as stiffness, viscosity, and diffusivity, can be precisely tuned, enabling the investigation of cellular responses to individual mechanical cues as well as their combined effects [154,155,156,157,158]. Polymeric biomaterials are engineered to respond to specific external signals reversibly. When the appropriate external stimulus is applied to the surface, cells can be detached without the need for enzymatic digestion or mechanical methods [159,160].

Bulk and surface properties have different effects on stem cell fate: the surface of a polymeric material is the first part that comes into contact with the cells, and the quality of the adhesion process affects the other cell interaction mechanisms. Cell adhesion behaviour differs between cells cultured on uniform 2D substrates and those on anisotropic surfaces with micro- or nanopatterned geometries [161,162,163]. Xie et al. categorised the physical properties of biomaterials into static cues, such as topography, elasticity, and ligand presentation, and dynamic cues, including responsive and self-regulating signals [164].

However, only a limited range of biomaterials with dynamic mechanical properties has been developed so far. The impact of these materials on cellular behaviours has been gradually revealed. Depending on how mechanical stimulation is applied to the cells, dynamic mechanical interactions can be classified into stimulus-responsive and self-regulated cues [165,166].

In this context, a successful biocomposite that has advanced towards clinical application is Integra^®^ Skin—an FDA-approved bioengineered skin substitute composed of collagen I, glycosaminoglycans, and a thin membrane of silicone that serves as a scaffold for dermal regeneration in cases of deep burns, chronic wounds, and reconstructive surgeries [167]. Its successful outcomes have extended its use to complex scalp reconstructions and other challenging wound environments [168].

### 2.3. Biodevices

The different mechanical stimuli sensed by cells in dynamic conditions are analysed by the use of biodevices such as microfluidics and organ-on-a-chip systems, which allow for the precise mimicry of a single “dynamic” functional unit of an organ in conditions closely resembling the in vivo environment (Table 2). In this manner, it is possible to examine the dynamicity with which these processes occur. The dynamism of physiological processes is a key to understanding how various mechanical stimuli can affect the cells.

A representative example is the shear stress generated by blood flow when hemodynamic forces encounter endothelial cells. Experiments in macroscale systems, such as parallel plate flow chambers, have demonstrated that fluid shear stress influences endothelial cell proliferation, migration, permeability, morphology, and gene expression [169]. Improved insights into these complex processes were made possible thanks to microfluidics, which is a transformative tool in the field of mechanobiology.

**Table 2:** Classes of biodevices involved in the study of mechanobiology.

Biodevices	Description	Advantages	Disadvantages	Applications	References
Microfluidics 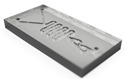	A technique that employs submillimetre-scale fluidic channels to precisely regulate the dynamic cellular microenvironment.	Mimics physiologic and pathophysiologic conditions with high spatial and temporal resolution.	May not fully capture the complexity of in vivo environments.	- *Microscale Tissue Engineering:* utilising microfluidics to simulate hepatic sinusoids.	[170]
				- *Bone Tissue Engineering*: generation of microdroplets based on alginate, collagen, and chitosan to simulate the ECM and enable osteogenesis.	[171]
				- *Simulation of Tumour Microenvironment*: microfluidic devices to generate compressed tumour spheroids to assess tumour cell migration, invasion, and immune response under biomechanical stimuli.	[172]
				- *Characterisation of cells*: using microfluidics with a constriction channel and planar electrodes to study mechanical and electrical characteristics of normal and hybridoma cells label-free.	[173]
Organ-on-a-Chip 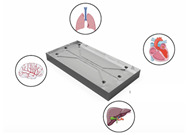	A bioengineered device that exploits advanced microfluidic systems to simulate organ functions, enabling the modelling of physiological processes at the tissue and organ levels. The simplest system is made by a single microfluidic channel; more complex designs feature two or more microchannels housing different cell lines.	Mimics tissue-specific dynamic microenvironment emulating human physiological processes; is less expensive than animal models.	Less experimental longevity than animal models; less throughput and scalability than 2D cell culture.	- *Modelling of Musculoskeletal (MSK) tissues* in vitro: using organ-on-a-chip to reproduce the microenvironment of MSK to study musculoskeletal disorders and evaluation of the toxicity of drugs and nanotherapy.	[174]
				- Tumour *Modelling*: biomaterials-organ-on-chip system to emulate the mechanistic events of the tumour metastatic cascade.	[175]
				- *Study of Viral Infection*: microvessel-on-a-chip preparation to establish the effects of NS1 protein of the dengue virus on the cell mechanics.	[176]
				- *Heart-on-a-Chip*: use of organ-on-a-chip with cardiac cells to model cardiovascular diseases and study potential toxic effects of drugs on the cardiac tissue.	[177]

#### 2.3.1. Microfluidics

Microfluidics is a technique that utilises submillimetre fluid channels to enable precise control over the cellular microenvironment, thereby mimicking physiologic and pathophysiologic conditions and monitoring the dose–effect response of a drug [178]. Moreover, by engineering microscale devices, it is possible to simulate conditions such as shear stress, compression, and stretching with high spatial and temporal resolution (Figure 2B). Moreover, the integration of microfluidics with live-cell imaging and advanced biosensors facilitates the real-time monitoring of cellular responses, providing novel insights into the interplay between mechanical forces and biochemical signalling [179,180]. This awareness is advancing the understanding of complex processes, such as tissue development and cancer metastasis, paving the way for innovative therapeutic strategies [181].

Importantly, microfluidics has been widely applied in organ-on-chip technology, revolutionising the field of mechanobiology by providing highly biomimetic platforms to study the mechanical and biochemical dynamics of tissues and organs [182].

#### 2.3.2. Organs-on-Chips

Organs-on-chips simulate the specific functions of organs by culturing living cells within continuously perfused, microscale chambers, enabling the modelling of physiological functions of tissues and organs [183]. Here, the goal is not to replicate entire organs but to recreate minimal functional units that mimic tissue- and organ-level behaviours [184]. The simplest system consists of a single microfluidic chamber containing a single type of cell line (e.g., hepatocytes or kidney tubular epithelial cells). More complex designs feature two or more microchannels housing different cell types, connected by porous membranes that recreate interfaces between distinct tissues, such as the blood–brain barrier [185]. These devices can integrate physical forces, such as fluid shear stress and mechanical compression, allowing for the analysis of organ-specific responses, including the recruitment of circulating immune cells, even in the presence of drugs, toxins, or other environmental stimuli [186]. The combination of microfluidics and organ-on-a-chip technology holds great promise for advancing drug discovery, personalised medicine, and the development of therapeutic strategies targeting mechanobiological pathways [185].

### 2.4. Other Systems Supporting Mechanobiological Applications

In addition to the tools mentioned above, implied in investigating cell–environment interactions and mimicking the dynamic nature of biological processes (respectively, biomaterials and biodevices), other conditions may also contribute to advancing mechanobiological research.

#### 2.4.1. 3D Printing

**Figure 3:**
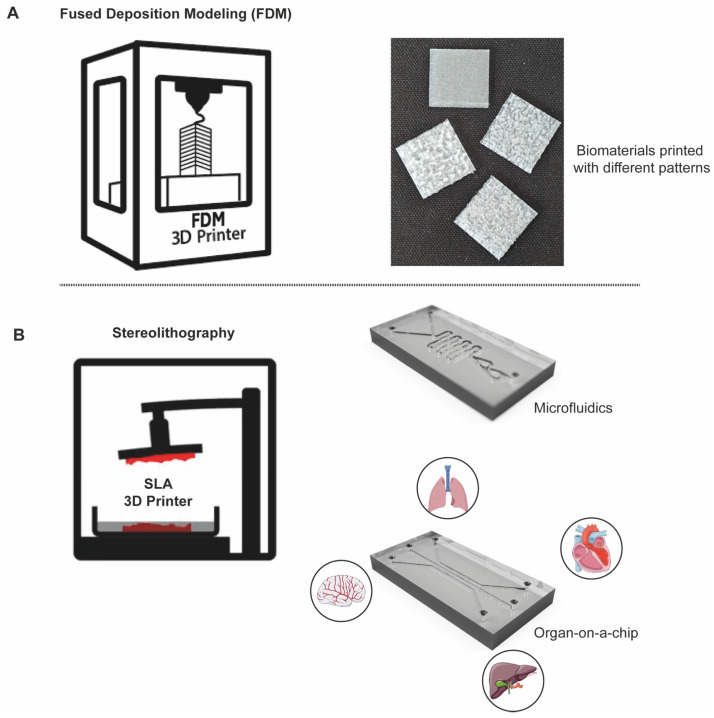
Scheme representing two different 3D printing systems. (**A**) Fused deposition modelling (FDM) is an additive manufacturing (AM) technology based on a liquefied filament that is then extruded through a nozzle. (**B**) Stereolithography (SLA) uses ultraviolet (UV) light to cure (harden) liquid resin layer by layer, building a 3D object. Among its applications, it is used to print biodevices for microfluidics and organ-on-a-chip systems.

The field of mechanobiology has taken advantage of 3D printing, as it provides versatile tools to investigate both the physical forces and mechanical properties of cellular behaviour. To this aim, 3D printing enables the precise fabrication of biomimetic structures that closely replicate the mechanical and architectural properties of native tissues. Among the seven 3D-printing technologies, the two most common are fused deposition modelling (FDM) and stereolithography (SLA) [187] (Figure 3). Of note, FDM is a 3D-printing method that composes objects by melting and extruding thermoplastic (or other materials) filament layer by layer. It is widely used for rapid prototyping, printing custom implants, tissue scaffolds, and medical devices using biocompatible thermoplastics like polylactic acid (PLA), polycaprolactone (PCL), and polyetheretherketone (PEEK) [188,189,190,191] (Figure 3A). SLA is a 3D-printing process that uses a UV laser to cure liquid resin into high-detail solid layers. It is commonly used for creating precise prototypes, high-precision anatomical models, custom dental and surgical guides, and microfluidic devices [192,193] (Figure 3B).

One of the key applications of 3D printing is the fabrication of artificial scaffolds and ECMs, which can be designed with complex geometries and controlled porosity to closely mimic the native ECMs. These structures provide both mechanical support and a three-dimensional microenvironment for cell growth, enabling the study of the effects of precisely customised parameters, such as stiffness, elasticity, and surface topography, on cell adhesion, proliferation, differentiation, and migration [194]. There are many advantages to 3D printing over traditional chemical fabrication techniques of biomaterials: 3D printing provides unprecedented spatial control, is highly customisable, and is highly compatible with automation and digital control, allowing researchers to program specific patterns, such as flow channels.

Materials such as PCL and PLA have been effectively used to create scaffolds for bone tissue engineering [195]. Additionally, 3D printing facilitates the development of controlled drug delivery systems, enabling the precise release of therapeutic agents, such as anti-inflammatory drugs or growth factors [196]. This is particularly relevant in mechanobiology, where localised delivery can modulate cellular and tissue responses to mechanical stimuli [197,198].

Furthermore, 3D printing is increasingly utilised in the production of microfluidic devices due to its speed, accuracy, cost efficiency, and capacity to generate geometrically complex structures [199,200,201,202]. A library of parametric components was developed for designing microfluidic devices for 3D printing, called Flui3D, thereby offering the ability to construct multilayered devices, enhancing accessibility and customisation for mechanobiology researchers [203]. Lastly, bioprinting, an advanced form of 3D printing, represents a transformative technology in regenerative medicine, as it allows the printing of tissues with unprecedented precision, where instead of “ink”, there are cells resuspended in the matrix and/or media, which is called bioink [204]. By utilising bioinks composed of living cells and supportive biomaterials, bioprinting enables the layer-by-layer deposition of three-dimensional architectures that closely replicate native tissues, offering valuable insights into how cells organise and respond within mechanically relevant 3D environments [205].

#### 2.4.2. Microgravity Systems

As mentioned above, the forces exerted on the cells of the body are different. Indeed, the human body, in addition to being subject to the forces noted before, is also affected by the forces applied by the surrounding environment. Notably, the gravity of the Earth has significant effects on the cells of our body in terms of structure and function [206]. Additionally, studies conducted under microgravity conditions have highlighted the critical role of gravitational forces on cellular behaviour [206,207,208,209,210,211]. For this, it is necessary to study the effects on cells under different conditions of gravity, giving rise to a new and emerging interest: microgravity. Microgravity is a condition in which gravity is very low or absent, as in space. This has a significant impact on the human body, causing various physiological changes [211,212].

Microgravity has profound effects on mechanobiology: in the absence of gravity, cells and tissues experience a significant reduction in mechanical loading, which disrupts normal mechanotransduction. This unique environment provides a valuable platform for studying how cells, tissues, and organisms adapt to altered mechanical conditions [208,210].

Microgravity-like conditions are simulated through clinorotation (where cells are rotated about an axis at such a slow rate that centrifugal force is so small as to be discounted) or random positioning (rotates biological samples along two independent axes) [213,214]. This enables controlled studies of cellular and tissue-level responses, providing new opportunities for drug testing, regenerative medicine, and understanding the interplay between mechanical forces and biological systems in extreme environments [213,215,216]. Under microgravity conditions, the lack of mechanical cues affects cellular behaviour, including changes in cytoskeletal organisation, gene expression, and protein synthesis [208]. For example, bone and muscle cells are susceptible to reduced mechanical loading, leading to bone resorption, muscle atrophy, and impaired tissue regeneration [217]. Bone loss in the absence of gravity was explored by Wubshet et al., who found that microgravity reduced YAP expression, whereas pressure increased it. The restoration of YAP provides insights into the influence of microgravity on the mechanical properties of bone cells and the impact of compressive pressure on cell signalling [208].

#### 2.4.3. Advanced Mechanobiology Platforms: BioMEMS, Microindentation, Micropipette and Bioreactors

Biological micro-electro-mechanical systems (BioMEMS) have emerged as valuable tools for investigating microfluidic systems, particularly those involving electrically active channels (e.g., mechanosensitive Piezo1). Notably, recent advancements have led to the development of magnetic polymer composites specifically designed for BioMEMS-based biomedical applications, including miniaturised drug delivery systems [218].

Another essential set of techniques for studying the mechanical properties of cells includes microindentation and micropipette aspiration (MPA) systems.

MPA involves the application of a controlled suction pressure via a micropipette to aspirate part of the cell membrane, allowing the quantification of mechanical parameters such as cortical tension and viscosity [219].

Conversely, microindentation involves pressing a calibrated indenter, such as an atomic force microscopy (AFM) tip or microscale probe, into the surface of a cell or tissue to measure its mechanical response, thereby providing critical data on stiffness and elasticity [220]. These properties are fundamental to understanding how cells respond to mechanical stress. A recent 2024 study by Boot et al. introduced a microfluidic platform that replicates MPA, enabling high-throughput mechanical phenotyping of multicellular spheroids and linking cellular mechanical characteristics to metastatic potential in cancer models [221].

Bioreactors also play a significant role in the field of mechanobiology. They are engineered systems that provide controlled environments for studying and manipulating biological processes. In mechanobiology, bioreactors are designed to apply mechanical stimuli, such as tension, compression, or shear stress, to cells or tissues, enabling researchers to investigate how mechanical forces influence cellular behaviour and tissue development [47,222,223,224,225,226].

#### 2.4.4. Computational Modelling in Mechanobiology

A complementary and transformative role in mechanobiology is occupied by computational modelling, enabling researchers to simulate, predict, and interpret the complex interactions between mechanical forces and biological systems. In tandem with experimental fabrication, computational modelling provides a powerful, complementary toolkit for scaffold and device design.

Finite-element analysis (FEA) is widely used to predict and optimise the mechanical behaviour of 3D-printed scaffolds—guiding porosity, architecture, and material–property selection under physiological loading conditions [227]. For instance, FEA-based studies have simulated stress distributions in PCL and PLA scaffolds to correlate pore geometry with scaffold stiffness and fatigue performance [228] and to assess endurance life under cyclic loading [229].

Computational mechanoregulation models integrate FEA with tissue-differentiation algorithms, thereby forecasting patterns of cell-mediated matrix deposition in biomimetic scaffolds for bone and cartilage tissue engineering [230]. Beyond single-scale analyses, multiscale simulations link molecular-scale dynamics with tissue-level mechanics, enabling the exploration of mechanotransduction pathways and emergent behaviours across scales [231].

Approaches such as multilevel finite element (FE^2^) embed microscale constitutive models within macroscale finite-element meshes to capture history-dependent material responses [232].

Soft-tissue multiscale models further demonstrate coupling between cell adhesion kinetics and tissue-level stresses, thereby predicting morphogenetic and homeostatic processes in vascular and musculoskeletal systems [233].

These in silico tools facilitate extensive parameter sweeps and hypothesis testing before fabrication, accelerating optimisation and reducing experimental burden [234]. Collectively, the integration of FEA and multiscale modelling with 3D printing underscores the interdisciplinary nature of mechanobiology—bridging experimental and computational realms to design, interpret, and refine mechanobiological systems in silico.

## 3. Mechanotransducer Proteins as Design Targets for Biomaterials

Internal stresses may trigger mechanotransduction pathways through proteins and receptors that detect mechanical inputs and convert them into signalling events, leading to different biological responses. The proteins involved in this process are called mechanotransducer proteins. These proteins can convert mechanical signals into chemical signals; i.e., upon a stimulus, the protein is phosphorylated or dephosphorylated, thus changing its subcellular localisation [20,235,236]. Different mechanotransducer proteins are involved in transduction pathways with YAP and TAZ serving as key regulators [8]. For instance, integrins act as upstream mechanotransducers—through integrin–FAK/Src signalling, they regulate YAP/TAZ activity, making integrin engagement itself a critical design target for biomaterials [21,237].

The YAP (Yes-associated protein) and TAZ (transcriptional coactivator with PDZ-binding motif) are mechanosensitive transcriptional coactivators that function as master regulators of mechanotransduction, downstream of the Hippo pathway. They are critical for cellular response to mechanical stimuli, as they are involved in many biological processes (proliferation, differentiation, growth, and metabolism). The activity and the subcellular localisation of YAP and TAZ are finely regulated by several factors, such as mechanical stimuli (i.e., ECM stiffness, cytoskeleton deformations, focal adhesions) [238], biochemical signals (growth factors or ATP levels through the activation of AMPK), interactions with other proteins (14-3-3 or angiomotin proteins) [164], and the Hippo pathway [239,240].

**Figure 4:**
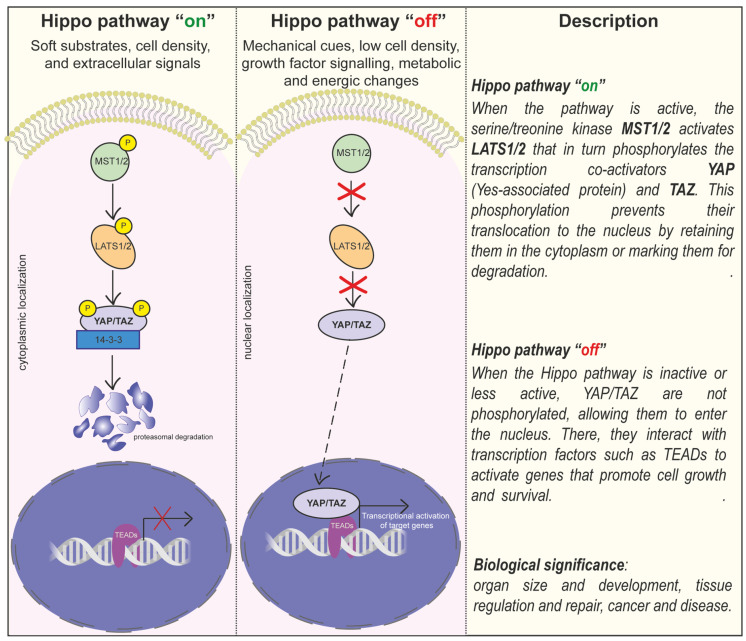
Overview of Hippo pathway and YAP/TAZ mechanotransduction. When the Hippo pathway is activated, the mammalian STE20-like protein kinases 1/2 (MST1/2) phosphorylate and activate large tumour suppressor kinases 1/2 (LATS1/2). LATS1/2, in turn, phosphorylates YAP/TAZ, inhibiting their role by “blocking” them in the cytoplasm and, consequently, degradation. When the Hippo pathway is deactivated, unphosphorylated YAP/TAZ translocates to the nucleus, whereby they promote the transcriptional activity of the transcription factors TEAD1/2/3/4 to regulate the transcription of genes associated with the cell-fate decision, such as cell proliferation, survival, and differentiation. Moreover, this pathway is known to be strictly regulated by various stimuli; for example, high ECM stiffness promotes the transcriptional activity of YAP/TAZ–TEAD.

The activity of YAP/TAZ is closely related to their localisation within the cell: when inactive, the phosphorylated YAP/TAZ are mainly located in the cytoplasm; when the cell senses mechanical stimuli, YAP/TAZ are dephosphorylated and translocated to the nucleus, where they interact with transcription factors to regulate gene expression [241]. Once in the nucleus, YAP/TAZ influences the expression of a wide range of genes together with DNA-binding transcription factors such as TEADs (TEA domain family members) [242]. Mechanical stimuli, such as substrate stiffness, promote F-actin polymerisation and stress fibre formation, prompting YAP/TAZ translocation into the nucleus to activate gene expression programs that regulate proliferation, differentiation, and survival. On the other hand, under normal conditions, soft substrates or mechanical inhibition activate the Hippo pathway, leading to phosphorylation and the cytoplasmic sequestration of YAP/TAZ [243] (Figure 4).

Given their essential role in cells, it is clear that dysregulation leads to different pathological conditions, depending on the context. YAP/TAZ activation is common in many types of solid tumours, where they promote cancer stem cell formation, growth, metastasis, and resistance to chemotherapy. Specifically, in HPV-negative head and neck squamous cell carcinoma, YAP/TAZ activation may be triggered by both genetic alterations and Hippo-independent mechanisms [244]. YAP/TAZ activation can also be caused by ECM stiffness, leading to tumour cell growth and invasiveness [101].

YAP/TAZ is involved in the pathogenesis of fibrosis, including liver, kidney, and lung fibrosis [245]. Matrix stiffness, which increases fibrosis, leads to the increased activation of YAP/TAZ in fibrotic cells [245,246,247,248]. An excellent work published a few months ago by Warren et al. showed that YAP/TAZ promotes the proliferation and secretion of ECM proteins in lung fibroblasts, creating a positive feedback loop that keeps pathological mechanotransduction active [249].

While it is well established that increased substrate stiffness generally promotes the nuclear localisation and activation of YAP/TAZ, recent studies have revealed a more nuanced and context-dependent behaviour, leading to ongoing debates in the field [250,251]. For instance, Basilico and colleagues demonstrated that by applying a low-frequency electrical field on MSCs, YAP was sequestered to the cytoplasm even though on a stiffer hydrogel. In contrast, cells on softer gels exposed to the same electrical field showed nuclear YAP [252]. In addition, there is evidence suggesting that YAP retains its nuclear transcriptional activity even under low mechanical tension [253,254,255]. These studies demonstrate that certain conditions can reverse the anticipated stiffness-dependent YAP/TAZ response, highlighting that YAP/TAZ activation in response to biomaterial stiffness is not universally predictable but rather is modulated by multiple factors, including cell type and the integration of diverse signalling pathways.

The misregulation of YAP/TAZ has also been found in laminopathies, where mutations in lamins lead to the constitutive nuclear localisation of YAP/TAZ [256]. The downstream effects of mechanotransducer proteins, such as YAP/TAZ and lamin A/B1, should be carefully considered in the design of novel biomaterials, as their activity emerges as a critical molecular indicator of how cells interpret and respond to their physical microenvironment. It is therefore necessary to tailor biomaterials for specific biological and therapeutic applications to achieve greater precision and efficacy, allowing for the exploration of biomaterial-based systems that mimic the functions of mechanoproteins [257]. For instance, by varying hydrogel crosslinking and thus substrate stiffness, it is possible to vary the subcellular localisation of mechanoproteins such as YAP/TAZ and lamin A/B1. Torres et al. have demonstrated that in hMSCs, nuclear YAP1 was localised in the nucleus on stiffer hydrogel. The composition of nuclear lamina was analysed, and researchers found that the lamin A/B1 ratio was significantly greater on the stiffer substrate, suggesting that the mechanosensitivity of cell adhesions and cell spreading with increased substrate stiffness is propagated to the nucleus of hMSCs cells [258,259].

Moreover, lamin A expression increases in a logarithmic mode in response to the rigidity, increasing in stiffness between 2 and 18 kPa. In this context, the rigidity of the substrate was investigated. It has been demonstrated that stem cells can migrate to stiffer substrates (durotaxis) in response to a certain gradient of rigidity [259].

Piezo1 is a mechanosensitive ion channel that responds to mechanical stress by allowing calcium (Ca^2+^) influx into the cell, leading to the activation of YAP/TAZ, thus acting as a critical sensor for mechanosensing. Piezo1 significantly contributes to the coordination of cell functions in both physiological and pathological conditions. In the heart valve, Piezo1 regulates YAP1 smooth muscle progenitor cells; in bone, it can respond to fluid shear stress and ECM stiffness signals to activate the NFAT/YAP1/β-catenin pathway in a Ca^2+^-dependent manner [260]. In endothelial cells, shear stress mediates Piezo1 activation and regulates vascular development, while its dysregulation can lead to vascular pathologies [261]. Indeed, the different expression levels of Piezo1 affect cell migration: it is strongly enhanced if overexpressed, while it is reduced in KO cells [262]. Piezo1 is also involved in cancer cell growth and metastasis [263,264].

Another mechanosensitive protein is the TWIK-related potassium channel, which regulates the flow of potassium in response to mechanical stimuli, gaining importance in various fields of mechanobiology as it responds to mechanical and osmotic changes, specifically in cardiovascular systems at both the heart and vascular levels [265]. In this circumstance, biomaterials with tuneable stiffness, such as hydrogels with controllable crosslinking densities or nanofibrous scaffolds with adjustable elasticity, have been engineered to modulate Piezo1 activity, promoting osteogenesis in bone tissue engineering or guiding stem cell differentiation. Similarly, TWIK-related channels, which regulate potassium flux in response to stretch or osmotic changes, are attractive targets in cardiovascular applications [266].

Electroconductive polymers, soft elastomers, or piezoelectric materials can be integrated into vascular or myocardial patches to mimic physiological mechanical cues and restore ion channel function in damaged tissues [267,268,269].

**Figure 5:**
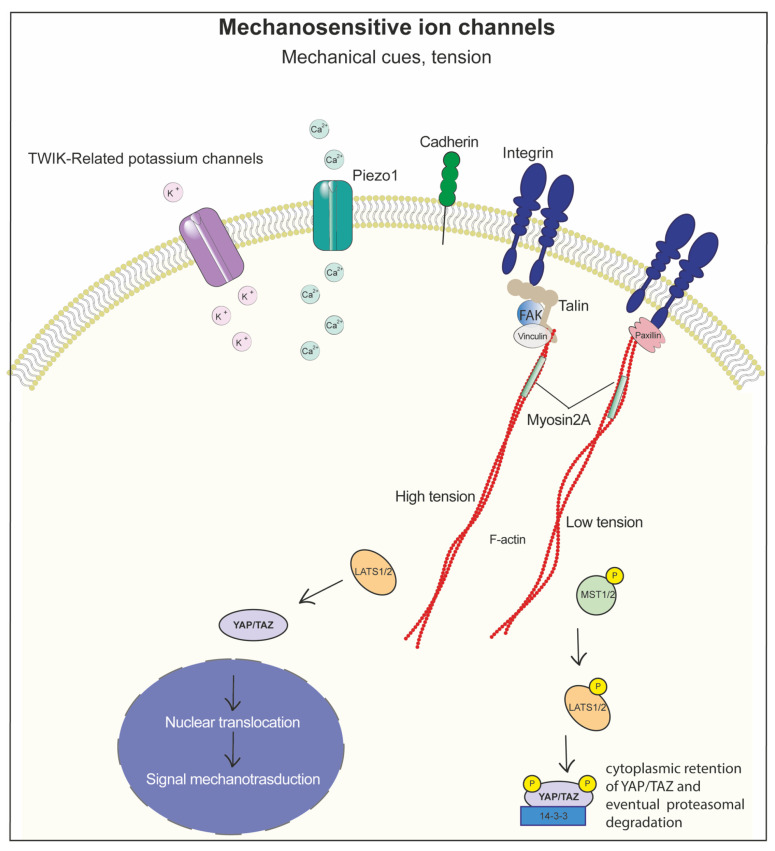
Overview of the mechanosensing pathway. The scheme represents the different membrane proteins involved in the perception of mechanical stimuli. The variation in ECM stiffness, mechanical stimuli, and extracellular signals may activate mechanosensitive ion channels such as TWIK-related potassium channels and Piezo1, and adhesion receptors such as integrin and cadherin, which in turn interact with cytoskeletal proteins (talin, vinculin, paxillin, focal adhesion kinase), linking F-actin filaments, thereby transmitting the tensions inside the cells.

Modulating the proteins involved in the mechanosensing process is critical for targeting diseases associated with their dysregulation (Figure 5). Biomaterials engineered to alter cytoskeletal tension or focal adhesion dynamics can exert direct control over the downstream signalling pathways governed by these proteins. Targeting these mechanosensors and mechanotransducers through material stiffness, the matrix of biomaterials with different physico/chemical properties, and surface topography allows for the calibration of cell behaviour, enabling precision control in tissue engineering, regenerative medicine, and mechanopharmacology.

Collectively, the integration of biomechanical design principles with advanced materials enables the precise manipulation of Piezo1, TREK channels, and cytoskeletal regulators, paving the way for more effective biomimetic strategies in regenerative medicine, disease modelling, and mechanotherapeutics [270,271,272,273,274].

## 4. Inside the Tension: How Organelles Sense and Respond to Force

As the plasma membrane, organelles may sense mechanical cues through membrane deformation, by the alteration of the contact sites, through the cytoskeleton, or by altering the intraluminal flow [275]. The organelles contained in the cell are largely interconnected and respond to mechanical stimuli by adapting their structure and function; this is fundamental for various processes, such as migration, growth, and differentiation [276,277].

### 4.1. Subcellular Organelles as Targets and Mediators of Mechanobiology

It is well known that cellular organelles, in addition to performing their canonical functions (i.e., producing energy for mitochondria, sorting of proteins for Golgi apparatus, protein synthesis for endoplasmic reticulum, etc.), perceive mechanical cues, thereby influencing cellular functions in response to mechanical stress as they are associated with the cytoskeleton [275].

**Figure 6:**
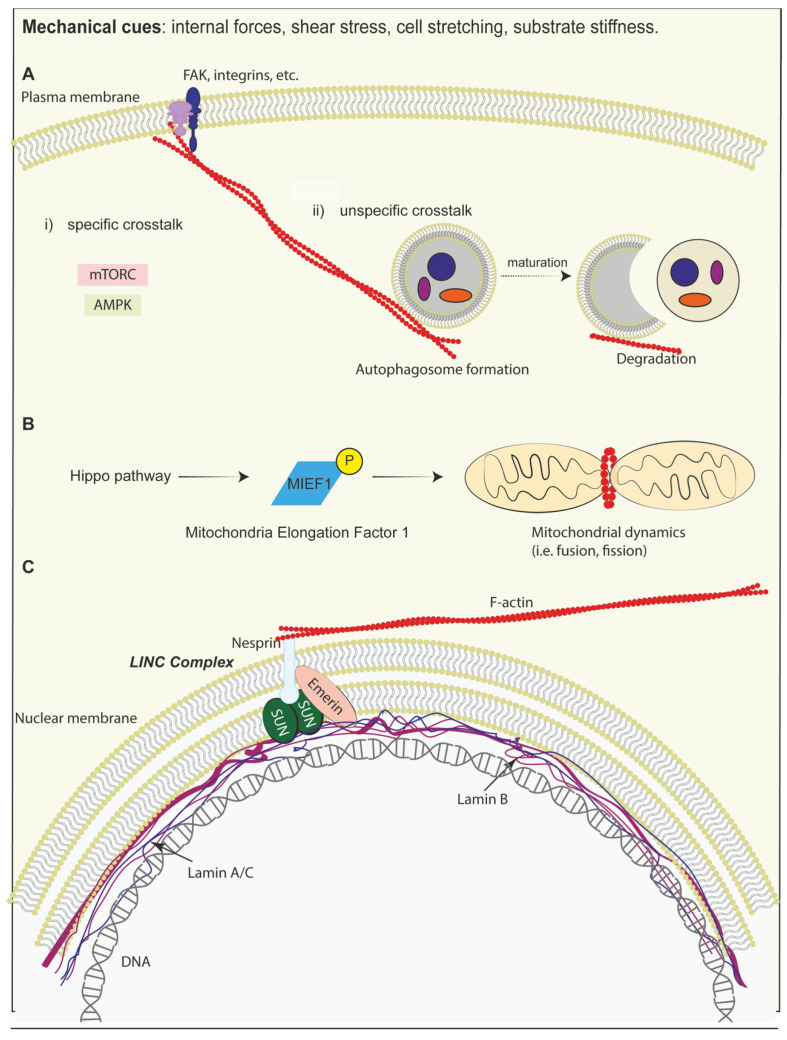
Mechanobiology of cellular organelles. (**A**) Schematic illustrating the interplay between mechanobiology and autophagy. Autophagy may be triggered via (i) specific crosstalk, or via (ii) unspecific crosstalk. In cases of unspecific crosstalk, mechanical cues regulate cellular tension and cytoskeletal architecture, triggering signalling pathways that modulate autophagic activity for maintaining cellular homeostasis. (**B**) Mitochondrial dynamics is strictly correlated to mechanical cues: a downstream effector of the Hippo pathway, MIEF1, is involved in the fusion/fission of mitochondria. (**C**) The nucleus, often regarded as the “command centre”, is not only deputy for DNA replication but also a dynamic structure that responds to mechanical stress. Mechanical forces can alter the organisation of the nucleoskeleton, affecting chromatin structure and gene expression. Moreover, the tension transmitted through F-actin is propagated to the nucleus via the LINC complex.

External forces may influence mitochondrial dynamics, as mitochondria respond to external forces by influencing mitochondrial dynamics. Indeed, mitochondrial fission (i.e., the process through which one mitochondrion is divided into two) nowadays is considered a mechanoresponsive event [275,278,279]. Surfaces may alter cell shape and cytoskeletal tension, regulating mitochondrial distribution and respiration (known as MIME, mitochondrial mechanotransduction), thereby linking physical confinement to cellular metabolic states [277]. It has been demonstrated that the mechanoproteins YAP/TAZ transcription cofactors are regulated by mitochondrial dynamics based on different mechanical stimuli. ECM stiffness and applied forces regulate mitochondrial dynamics, particularly fission, through the phosphorylation of the mitochondrial elongation factor (MIEF1). Mitochondrial fission, in turn, regulates YAP/TAZ, thereby influencing cell proliferation, lipid metabolism, and oxidative stress response [280,281]. These effects were observed across various cell types, both in vitro and in vivo, suggesting a unifying role of mitochondria in mechanotransduction and cellular homeostasis [277]. Moreover, electroconductive biomaterials, such as graphene-based scaffolds, have been reported to enhance mitochondrial activity and ATP production, promoting myogenic and neurogenic differentiation in stem cells [282,283] (Figure 6B).

The nucleus is a mechanosensitive organelle: directly applied forces facilitate YAP nuclear translocation by lowering the mechanical resistance of nuclear pores to molecular transport. In stiff environments, cells form a mechanical linkage between the nucleus and the cytoskeleton (called LINC, linker of nucleoskeleton and cytoskeleton complex, which is a protein complex linked with both inner and outer membranes of the nucleus) [284,285]. In this context, cytoskeletal filaments, which are assisted by focal adhesion proteins outward, bridge the nucleus through different proteins, among which are nesprin 1/2, nesprin 3, and SUN protein. At the same time, lamin A/C provides structural support to the nucleus, enabling force transmission from focal adhesions to the nucleus [286,287] (Figure 6C). This leads to nuclear flattening and stretching of the nuclear pores, reducing their resistance to molecular transport and thereby enhancing YAP nuclear import [288].

Cells in an atypical environment respond to different external stimuli by the reorganisation of cytoplasmic organelles, too. It has been demonstrated that in various diseases, a phase transition from a solid-like to a liquid-like state or vice versa [52,289]. In this context, tissue-level mechanical transition leads to the deformation of cells and nuclei, resulting in the activation of an aberrant mechanotransduction pathway. Frittoli et al. have demonstrated that invasive breast cancer is characterised by the fluidification of tissues, leading to nucleus breaking and the release of genetic material into the cytoplasm [290]. Consequently, cells increase in nuclear stiffness and reorganise heterochromatin, leading to transcriptional reprogramming favouring malignant traits. Interestingly, the nuclear envelope protein lamin A is differentially expressed in response to the mechanical properties of the surrounding microenvironment: it is more abundant in stiff tissues and downregulated in softer ones [291,292]. Experimental findings indicate that stem cells cultured on rigid substrates exhibiting high lamin A expression preferentially undergo osteogenic differentiation. In contrast, those on softer matrices with reduced lamin A levels tend to adopt an adipogenic lineage [293,294].

The endoplasmatic reticulum can both perceive mechanical cues through its mechanosensitive channels and also be a target of the mechanotransduction signalling pathway [275]. The Golgi apparatus, a central hub in protein and lipid trafficking, also responds to changes in the cellular mechanical environment. Studies have shown that cytoskeletal disruption caused by soft substrates or a lack of adhesion can lead to Golgi fragmentation and impaired protein processing. Furthermore, biomaterials that modulate actin architecture, such as nanofiber scaffolds or surface micropatterns, can indirectly control Golgi positioning and vesicle trafficking, which are essential for polarised cell migration and secretion [295]. Collectively, these findings highlight the importance of rational biomaterial design not only in guiding extracellular interactions but also in remodelling intracellular organelle function. The exploration of the mechanobiology of subcellular organelles is crucial for understanding cellular processes in both physiological and pathophysiological conditions.

### 4.2. Mechanobiological Cues from Biomaterials Regulate Cell Metabolism

Based on the interaction between cells and biomaterials, the mechanotransduction pathway is activated, leading to alterations in the cytoskeletal network involving the propagation of tension towards the inner part of the cell, thereby conditioning cellular organelles and the nucleus.

By exploiting specific biomaterial characteristics, it is possible to drive the cell fate towards a specific purpose. Moreover, the properties of a material may influence the adsorption of proteins from the surrounding environment, which in turn can affect cell signalling and metabolism [296]. For instance, biomaterials composed of metal ions have a significant impact on osteoblast metabolism, as they act as enzyme cofactors involved in bone formation [296]. Calcium phosphate biomaterials can impact cell metabolism as they adsorb metabolites, such as amino acids, thereby influencing amino acid and energy metabolism pathways by altering the availability of cellular nutrients [297]. Moreover, the incorporation of metal ions into a biomaterial matrix can significantly change its mechanical behaviour: Stadter and colleagues showed that embedding silicate microparticles doped with Li^+^, Mg^2+^, Sr^2+^, or Zn^2+^ into a collagen scaffold not only released bioactive ions but also modified the scaffold’s stiffness and deformation profile [298].

The degradation of biomaterials can release regulatory metabolites, such as citrate and inorganic phosphate, which can affect intracellular metabolism [299]. Citrate incorporation enables precise control over a polyester’s initial stiffness and promotes gradual softening through ion binding and degradation, making these polymers ideal for dynamic, tissue-matching applications [300,301]. It has been demonstrated that hMSCs internalise citrate released from citrate-based biomaterials, increase energy production via oxidative phosphorylation, and inhibit glycolysis, resulting in increased intracellular ATP levels (this process is called energy homeostasis) and the promotion of osteogenic differentiation [301,302]. Cell–biomaterial interaction may also trigger an anti-inflammatory response. Chuying Ma and colleagues have observed that poly (ethylene glycol) hydrogel containing lactate reduces intracellular reactive oxygen species and improves survival [299,303]. Inorganic phosphate released from mineralised matrices containing calcium phosphate can be uptaken by hMSCs, increasing intracellular ATP and promoting osteogenesis via the release of adenosine as an autocrine/paracrine signalling molecule [299,304,305].

Moreover, biomaterials may affect epigenetics, such as histone de- or acetylation and methylation, thereby influencing cell behaviour [306,307]. In more detail, it has been demonstrated that microtopography is capable of reversibly inducing cell quiescence in hMSCs, which is characterised by a reduction in cell metabolism and proliferation [306]. Additionally, nanotopography, as influenced by nanotubes, has been shown to have a similar effect on the cell morphology of hMSCs [308]. Given the well-established dynamic regulatory influence of biomaterial cues on cellular functions, the potential effects of these cues on cell metabolism and their subsequent impact on modulated cellular behaviour may be a new approach to gaining a 360-degree view of biological processes.

#### Modulation of Autophagy by Mechanical Stimuli

Recent evidence in the literature has shown a link between mechanobiology and metabolic processes, particularly in relation to autophagy [309]. Autophagy is a cellular homeostatic mechanism employed for the recycling of damaged organelles and the turnover of proteins involved in cellular migration. However, recent studies have observed that this process is exploited by cells as an adaptive response to survive through unfavourable stresses [310]. In general terms, mechanical cues may affect the autophagic process in two ways: (i) via specific crosstalk between the cell signalling of mechanotransduction and autophagy proteins, i.e., mTORC and AMPK; (ii) via unspecific mechanisms through the cooperation of cytoskeletal elements [309,311,312] (Figure 6A). Namely, it has been demonstrated that haemodynamic shear stress can trigger the phosphorylation of signalling molecules related to the cytoskeleton, like integrins and focal adhesion kinases, and also membrane lipid raft-associated cytoplasmic proteins like p48 MAP kinases, which activate autophagic cascades. Indeed, the flow-induced activation of FAKs causes cytoskeletal rearrangement, leading to the formation of autophagosomal components. In cancer, the activation of the autophagic process is related to cancer resistance, as it facilitates the turnover of focal adhesion proteins, thereby promoting the survival and migration of cancer cells [313,314].

For this reason, targeting pathways related to the mechanotransduction of autophagy may be a successful approach for different diseases. Moreover, in other cell types, contrasting effects were observed on YAP/TAZ signalling in response to alterations in autophagy. The inhibition of YAP/TAZ correlates with the failure in the modulation of gene expression of myosin-II and consequent loss of F-actin stress fibres, leading to impairment in autophagosome formation [315]. On the other hand, low cell density induces YAP/TAZ activation in the nucleus, leading to autophagosome assembly and a feedback loop between autophagy and the Hippo pathway [316,317]. The correlation between cellular trafficking and mechanobiology was confirmed by chloroquine treatment, which induces the expression of YAP in cancer cell lines [318]. Interestingly, the autophagy process is strictly related to alpha-catenin, which is involved in linking cadherins and the actin cytoskeleton: YAP/TAZ activity is increased upon autophagy inhibition, while it is reduced by autophagy when alpha-catenin levels are high [315]. Vice versa, inhibition of the Hippo pathway, i.e., with high cell density, reduces the efficiency of the autophagic flux [319,320].

Intriguingly, bone regeneration can be achieved through the stimulation of autophagy, and tuning this process allows for the control of cell differentiation in tissue engineering [321,322]. To this aim, different biomaterials and structures were investigated. Nanotube (NT) structures have been shown to enhance mTOR-independent autophagy in osteoblasts more effectively than flat surfaces [323]. Zhang and colleagues demonstrated that gold nanoparticles (AuNPs) promote osteogenesis by activating autophagy, which is characterised by the upregulation of microtubule-associated protein LC3 and downregulation of sequestosome 1/p62 [324]. Moreover, studies on different structures and sizes of nanoporous anodic alumina have demonstrated that the autophagy pathway is orchestrated through the activation of LC3A/B, Beclin-1, Atg3, Atg7, and p62, influencing osteoclastic activity and leading to changes in the differentiation of bone marrow stromal cells [325]. Kaluđerović et al. reported that autophagy-dependent PI3K/Akt signalling plays a crucial role in osteoblast differentiation on titanium-based surfaces with rough topography [326]. This demonstrates that the interaction between nanotopography and cells holds the potential for various medical applications. Due to their versatility and dynamic properties, active biomaterials open new frontiers for understanding cell–matrix interactions and developing more physiologically relevant models for tissue engineering and regenerative medicine, paving the way for new frontiers of pharmacology.

## 5. Challenges and Future Directions in Mechanobiology

Despite notable advancements in engineering systems that mimic the complex mechanical environments of living tissues, several critical questions remain unresolved, and new opportunities are emerging [327].

A central challenge is the integration of dimensionality, dynamic feedback, and physiological complexity within in vitro models [328]. Many current systems successfully capture static or simplified aspects of tissue mechanics but fail to fully replicate the spatiotemporal heterogeneity of in vivo cues, e.g., cyclic transient stiffening during development or disease. Future research must address the standardisation and scalability of biomaterial platforms. The batch-to-batch variability in scaffold properties and lack of consensus on mechanical parameters (e.g., optimal strain rates for different cell types) hinder reproducibility and cross-comparison between studies.

Although numerous platforms have demonstrated substantial potential in both in vitro and in vivo studies, only a small fraction have advanced to clinical implementation. Even though biological systems are inherently complex, it is crucial to translate research findings into practical applications, which requires a careful balance between innovation, safety, and efficacy [329,330].

Tissue-engineered products frequently incorporate different elements of medical devices (e.g., scaffolds or matrices), biological materials (e.g., cells or growth factors), and pharmacological agents (e.g., sustained-release therapeutics), which results in their classification as “combination products” under regulatory frameworks. This multifaceted composition requires that they must satisfy the safety and efficacy requirements of each constituent part while also demonstrating the performance of the integrated product as a whole, thereby delaying clinical translation [331].

One of the main barriers to implementation is regulatory oversight. The European Medicines Agency (EMA) is responsible for evaluating, approving, and monitoring medicines within the European Union, ensuring the safety and benefit–risk balance of medicinal products, including concerns such as immunogenicity, treatment failure, and unintended tissue formation. The EMA has released a set of regulatory guidelines on the topics to be considered by companies involved in the development and marketing of medicines for use in the European Union (URL https://www.ema.europa.eu/en/human-regulatory-overview/research-development/scientific-guidelines/multidisciplinary-guidelines/multidisciplinary-cell-therapy-tissue-engineering, accessed on 8 June 2025). These guidelines must be followed for the marketing authorisation of a product to be issued.

The next decade of mechanobiology will likely be defined by efforts to build multiscale, dynamic, and immunocompetent models, integrate predictive computational tools, and overcome translational hurdles through interdisciplinary innovation [332]. These developments will accelerate the development of next-generation regenerative therapies and personalised medicine while also expanding our understanding of how mechanical forces influence biology.

By gaining insight into how cells and tissues react to mechanical stimuli, the developing field of mechanobiology helps us better understand several biologically essential processes, such as development, homeostasis, and the progression of disease, as well as how mechanical cues interact with biochemical signals. The comprehension of these aspects enhances the tailoring of biomaterials (both traditionally manufactured and 3D printed) to understand better how cells interact with various substrates as well as the understanding of dynamicity in pathological and physiological processes with the aid of biodevices. In this context, mechanotransducer proteins have emerged as potential targets for therapeutic interventions in various diseases [7,333,334]. According to this, disease treatments may be revolutionised in a few years through these innovative approaches.

Greater knowledge of mechanobiology—more especially, how cells interact with their surroundings and the outside world—will help develop more efficient regenerative medicine and personalised therapy strategies, opening the door to essential breakthroughs in biotechnology and healthcare.

## Figures and Tables

**Table 1:** Biomaterials for mechanobiology.

Biomaterials	Description	Advantages	Disadvantages	Applications	References
Scaffold 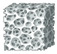 Hydrogel 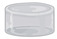 Film 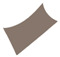 Nanoparticles ** 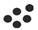 **	Engineered materials with specific physicochemical properties to influence cell behaviour. Structure design: films, scaffolds, hydrogels, nanoparticles	Customisable properties for distinct manipulative cues	May not fully mimic dynamic physiological processes	- *Bone regeneration*: stiff biomaterials mostly composed of collagen, graphene oxide and bioceramics	[56,57,58,59,60,61,62]
				- *Peridontal regeneration*: zirconia and hydroxyapatite improving the performance of dental materials	[63,64,65,66]
				- *Neurogenic differentiation*: softer biomaterials mostly composed of collagen, gelatin, hyaluronic acid, poly(butylene 1,4-cyclohexane dicarboxylate) (PBCE), and PBCE-based copolymer containing butylene diglycolate co-units (BDG50)	[67,68,69,70]
				- *Corneal regeneration:* collagen and thiol-functionalised collagen patch for vision restoration	[71,72,73]
				- *Adipogenic differentiation:* soft biomaterials (but slightly stiffer than those for neurogenic differentiation) composed of silk, collagen, hyaluronic acid, and polyethylene glycol	[74,75,76]
				- *Platform for mechanobiological research:* generation of engineered tissues based on flexible microfilaments on which it is possible to replicate mechanical properties of tissues	[77]
				- *Micropatterning*: precise surface structuring of biomaterials results in customised microscopic patterns/structures influencing cell response regarding growth, proliferation, differentiation, and polarisation	[78,79,80]
Micro- and Nanocomposites 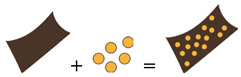	Engineered materials composed of a matrix combined with nanoscale components with one, two, or three dimensions less than 100 nm	Customisable properties for distinct manipulative cues	May not fully mimic dynamic physiological processes	- *Environmental and industrial applications*	[81,82]
				- *Antimicrobial properties:* food preservation, antiviral infections	[83,84,85,86]
				- *Piezoelectric-based bone regeneration:* hydrogel of oxidised chondroitin sulphate + amino-modified barium titanate nanoparticles (KBTO) promotes osteogenic differentiation	[87]
				- *Skin regeneration:* nanocomposites biomaterial such as nanofibers and nanoparticles offers a great potential for skin regeneration and wound care	[88]
				- *Cancer treatment:* the carbon/polymer nanocomposites as drug carriers	[89]
Smart Biomaterials 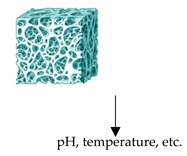 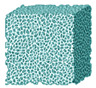	Innovative biomaterials that can modulate their properties and structures in response to external stimuli, allowing dynamic interaction with biological systems	High versatility and customisation of their properties, allowing the provision of specific cues to cells	Present an elevated complexity in design and fabrication with reduced stability and durability compared to other biomaterials	- *Soft Tissue Regeneration*: shape memory polymers (SMPs) of poly(glycerol dodecanedioate) acrylate (APGD) with different properties related to temperature	[90]
				- *Drug Delivery:* nanofiber mats of polycaprolactone and gelatin for mechanomodulate drug delivery under uniaxial mechanical stimulation	[91]
				- *Immunoactive biomaterials:* carragenine type λ binds to non-hydrolysed interleukin-8, promoting the differentiation of monocytes into macrophages	[92]
				- *Cancer treatment:* Au nanoparticles conjugate with pH-sensible aptamers that promote, at acid pH, the aggregation of nanoparticles and apoptosis of cancer cells	[93]

## Data Availability

Not applicable.
